# Granzymes in health and diseases: the good, the bad and the ugly

**DOI:** 10.3389/fimmu.2024.1371743

**Published:** 2024-04-05

**Authors:** Lavinia Cigalotto, Denis Martinvalet

**Affiliations:** ^1^ Laboratory of Reactive Oxygen Species and Cytotoxic Immunity, Department Biomedical Sciences, University of Padova, Padova, Italy; ^2^ Veneto Institute Of Molecular Medicine (VIMM), Padova, Italy

**Keywords:** granzyme, cytotoxic lymphocytes, cancer, inflammation, wound healing

## Abstract

Granzymes are a family of serine proteases, composed of five human members: GA, B, H, M and K. They were first discovered in the 1980s within cytotoxic granules released during NK cell- and T cell-mediated killing. Through their various proteolytic activities, granzymes can trigger different pathways within cells, all of which ultimately lead to the same result, cell death. Over the years, the initial consideration of granzymes as mere cytotoxic mediators has changed due to surprising findings demonstrating their expression in cells other than immune effectors as well as new intracellular and extracellular activities. Additional roles have been identified in the extracellular milieu, following granzyme escape from the immunological synapse or their release by specific cell types. Outside the cell, granzyme activities mediate extracellular matrix alteration via the degradation of matrix proteins or surface receptors. In certain contexts, these processes are essential for tissue homeostasis; in others, excessive matrix degradation and extensive cell death contribute to the onset of chronic diseases, inflammation, and autoimmunity. Here, we provide an overview of both the physiological and pathological roles of granzymes, highlighting their utility while also recognizing how their unregulated presence can trigger the development and/or worsening of diseases.

## 1 Introduction

Proteases represent a family of enzymes that hydrolyze the peptide bonds between amino acids, resulting in the proteolysis of proteins into smaller peptides or amino acids (1, 2). The activities of proteases are fundamental to cellular biology, as they take part in the regulation of several biological processes by modulating the structure, function, and fate of proteins. Through these activities, proteases indirectly influence cellular proliferation, differentiation, and angiogenesis, wound repair, inflammation, and apoptosis (3, 4). Independent of their role in maintaining tissue homeostasis, proteases also participate in the development of pathological conditions when they escape normal regulation. Extensive research has provided unequivocal evidence that proteases participate in cancer, rheumatoid arthritis, viral infections, neurodegenerative diseases, and immune disorders (1, 2, 5–7).

Among the diverse types of proteases, the members of the granzyme family of serine proteases are attracting new interest in the research community, due to their newly discovered extracellular roles as well as their participation in different physiological and pathophysiological processes. Since their discovery in the 1980s when they were originally terms granule-secreted enzymes, granzymes have been associated with the cytotoxic granule-mediated cell death pathways of cytotoxic T lymphocytes (CTLs) and natural killer (NK) cells, contributing, together with the pore-forming protein perforin, to the killing of infected or malignant target cells (8). Granzymes belong to the serine-protease family, and five human members have been identified: granzyme A (GA), granzyme B (GB), granzyme H (GH), granzyme M (GM) and granzyme K (GK) (9). Similarly, the mouse genome encodes numerous granzyme proteases (GA-G, K, M, and N) with cytotoxic potential, although they have different substrate specificity and function compared to their human counterparts (10). This is well illustrated as human GB cleaves human caspase 3 better than mouse caspase 3, and reciprocally mouse GB cleaves mouse caspase 3 better than human caspase 3 (11, 12). Importantly, human GB cleaves both human and mouse Bid, which is not true for mouse GB (11). While mouse GB is less efficient than human GB in activating Bid, both mouse and human GB are capable of initiating Bid-dependent mitochondrial apoptosis in intact cells (13, 14). Consistently, human adenovirus type 5, 100,000-molecular-weight (100K) assembly protein (Ad5-100K) specifically binds to human GB by interacting with both its catalytic pocket and an exosite outside the active site to inhibit human GB but not mouse or rat GB (15). A possible explanation for these differences might relate to exposure to species-specific pathogens and immune challenges, which allowed for the selection of new killing mediators (16). This difference in substrate specificity between human and mouse granzymes could partially explain some discrepancies in the vast literature on these proteases. Moreover, further research is needed to clarify how the 10 mouse granzymes functionally complement the restricted five human homologs.

Although their genetic sequences are almost 40% homologous, human granzymes possess unique substrate specificity, functions, and cell type expression, indicating the extremely complex and heterogenous context of their activities (17, 18). GB cleaves after aspartic acid residues, with a lesser preference for glutamic acid. GA cleaves after arginine or lysine, as does GK, its closest homolog. GM cleaves after the amino acid leucine or methionine while GH cleaves after the amino acid tyrosine or phenylalanine (19–23). Given the potent cleavage activity of the granzymes, their synthesis and storage processes are highly regulated to avoid accidental cytotoxicity of the killer cells. First, the translated preprogranzymes are directed to the endoplasmic reticulum (ER) where the signal sequence is removed, producing a proenzyme with an N-terminal dipeptide. In the Golgi apparatus, the addition of a mannose-6-phosphate motif addresses the proenzyme to the cytotoxic granules final compartment (24, 25). It is worth stressing that mannose 6-phosphate receptor-independent sorting has also been proposed (26). Indeed, earlier work using lymphocytes isolated from one patient with an autosomal defect in N-acetylglucosaminyl-phosphotransferase responsible for the phosphorylation of mannose, showed that about 30% of GA reached the cytotoxic granules (26). The fact that in these cells where mannose is not phosphorylated and, therefore, the mannose 6-phosphate receptor pathway is ineffective, GA is still transported within the cytotoxic granules indicates the presence of an alternative route to the cytotoxic granule independent of the mannose 6-phosphate receptor pathway (26). It is likely that this receptor- mediated trafficking increases the efficiency of granzyme delivery in the granules. However, more work is required to achieve a full understanding of this aspect of granzyme maturation. Once, in these specialized secretory lysosomes, cathepsin C-dependent proteolytic removal of the N-terminal dipeptide activates the granzymes that are then stored along with perforin bound to the serglycin proteoglycan. Indeed, it was reported that naïve NK cells and CTLs activated over 4 days from serglycin-deficient mice exhibit a profound reduction in cytotoxicity due to the reduced GB content in NK cells and reductions in both perforin and GB in these CTLs (27). These reductions in GB and perforin content were partly alleviated following IL-2–mediated activation of the NK cells or prolonged activation of the CTLs (27). Loss of serglycin also led to increased constitutive secretion of GB and virtually the absence of dense core granule in CTLs, pointing to a putative role of serglycin in cytotoxic granule biogenesis in addition to its granule storage function (27). In contrast, GA expression was unaffected by the loss of serglycin, suggesting its trafficking differs from that of GB (27). These results are in marked contrast with a previous report showing that loss of serglycin, although affecting the expression of GB and perforin, had no effect on the late cytotoxicity of mitogen activated CTLs (24). This apparent discrepancy can be explained by the fact that in this earlier study, killing was assayed after 21 hours, which likely reflected the death receptor pathway more than the faster acting cytotoxic granule pathway (24). Together, the acidic pH and the tight interaction with serglycin increase the packing of the granule while keeping both the granzyme and perforin inactive (28).

Traditionally, granzymes have been investigated in the context of cell death, focusing primarily on their intracellular roles. However, in recent years, this initial dogma has been revisited with new findings of granzyme presence in the extracellular milieu as well as their increased concentration in several diseases, suggesting other putative functions (29, 30). Indeed, numerous reports have supported the potential roles of granzymes in inflammation, autoimmunity, and age-related cardiovascular and pulmonary diseases (31). Therefore, granzymes should no longer be considered mere cytotoxic enzymes but rather studied in relation to the distinct roles they play intra- and extra-cellularly, exploring all their possible implications in health and disease. Here we review the current knowledge of granzyme functions, highlighting the processes through which granzymes contribute to cellular homeostasis as well as their negative contributions to disease progression. The topic of granzyme in health and disease has also been review recently by Nussing, S. et al. (32).

## 2 Granzymes in Health: The good

*Granzymes in homeostasis maintenance*

### 2.1 Cytotoxic functions in granule-mediated cell death

**Figure 1:**
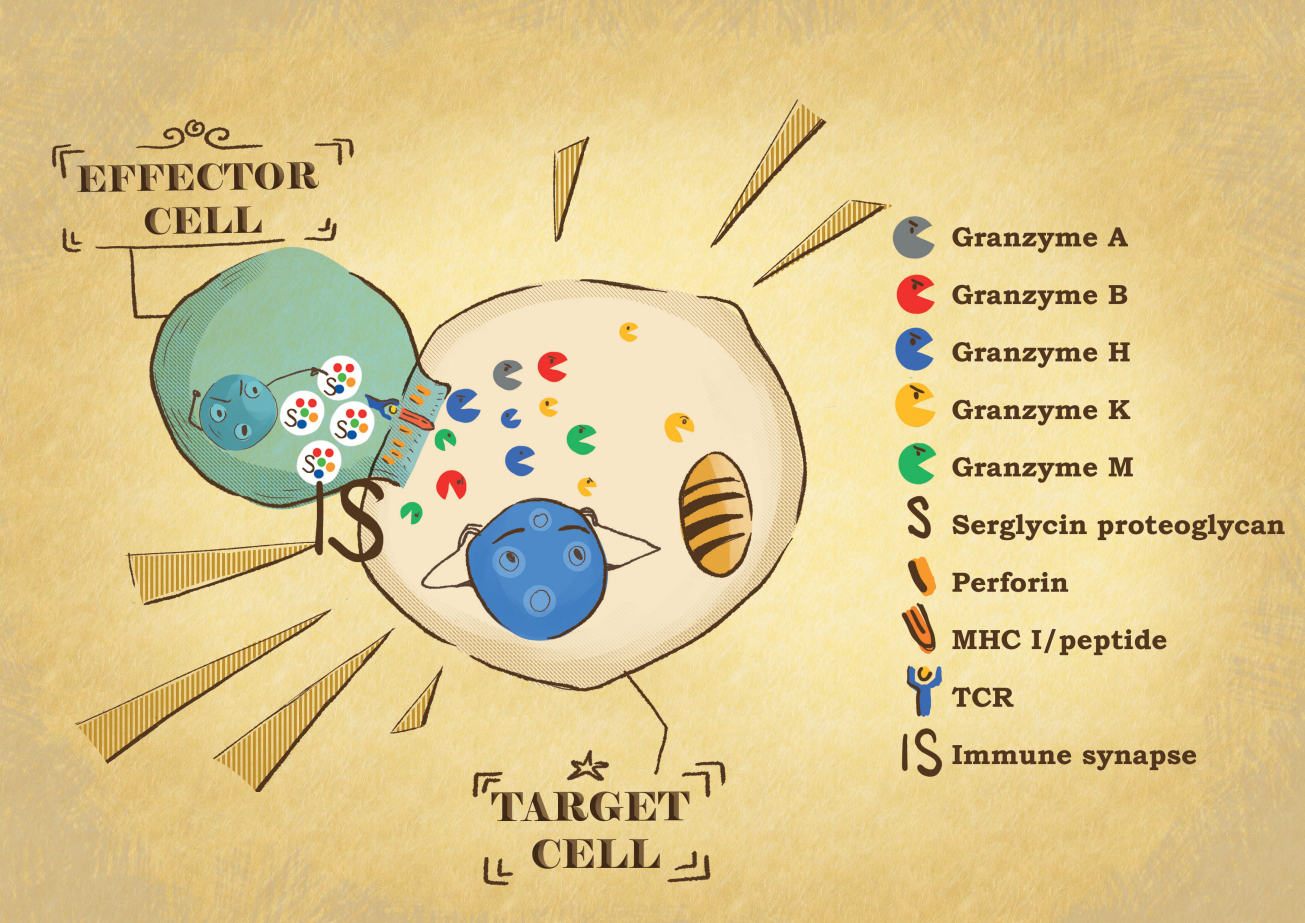
Effector: target cell conjugate formation. When the effector cytotoxic T lymphocytes or NK cells recognize their target cell, they form a tight conjugate separated by a narrow space called the immunological synapse (IS). By exocytosis, the effector cells release the content of their cytotoxic granules into the IS. Granzymes are delivered into the target cell by perforin. In the target cells, the granzyme proteases induce the death of the cells through different pathways, affecting different cell compartments.

A well-accepted role of granzymes is their participation as key mediators of target cell killing by NK cells and CTLs. Upon activation of the effector cells, cytotoxic granules move along microtubules and align at the immunological synapse (IS) that is formed between the effector and target cells. The membrane of the granule then fuses with that of the effector cell and releases its content in the synaptic space, from where perforin allows the granzyme to enter the target cell cytosol where it promotes rapid cell death (33) (**Figure 1**). The mechanism employed by perforin to deliver granzymes into target cells is still debated. The different models agree that perforin, a pore forming protein, oligomerizes in the membrane to form a pore through which granzymes are delivered. However, they disagree on the shape, size, and location of the pores. The first model supports the formation of the pore at the plasma membrane of the target cells. Using recombinant mouse perforin and site-directed mutagenesis, arginine 213 and glutamic acid 343 were found to be critical for the intermolecular interaction of perforin monomers and their ability to oligomerize (34). Furthermore, by X-ray crystallography and cryo-EM, the same group demonstrated that perforin is a key-shaped protein composed of an amino-terminal membrane attack complex perforin-like (MACPF)/cholesterol dependent cytolysin (CDC) domain upstream to an epidermal growth factor (EGF) domain, which is followed by a C2 carboxy-terminal sequence, that initiates the Ca^2+^-dependent binding to the membrane (35). During pore formation, the clusters of α-helices CH1 and CH2 of the MACPF unwind to form amphipathic β-strands that insert into the membranes (35). The C2 domain, by coordinating two Ca^2+^ ions in a pH neutral environment, binds strongly to the membrane and triggers this conformational change of the α-helices CH1 and CH2 (35). The resulting pores have a size distribution ranging from 50–300 A° with most of the pores being between 130–200 A° in diameter, which is large enough for granzyme delivery (35). This elegant structural characterization provides a clear mechanistic understanding of perforin oligomerization, but did not definitively demonstrate a unique occurrence at the target cell plasma membrane, as the data were compatible with perforin action at either the plasma or the endosomal membrane, as suggested by the alternative wound healing model (35). Another model questioned the cylindrical nature of the pore and suggests that perforin pores are formed by an incomplete arc-shaped protein oligomer able to rapidly induce the externalization of the phosphatidylserine at the protein oligomer-lipid interface (36). These arc-shaped perforin oligomers could be stabilized by the pf-80 anti-perforin antibody (36). One concern regarding the results supporting these two models is whether the dose of perforin used is physiologically compatible with what is delivered by cytotoxic lymphocytes. More recently, the perforin pore was investigated using atomic force microscopy analysis of ova-B16 target cells treated with purified perforin (37). On average, the formation of seven pores of about 594 ± 50nm to 476 ± 40 nm over a 5 × 5 µm^2^ area were observed (37). If the pore size is compatible with the previous pore size distribution, one must note that in their experiments 100% of the perforin-treated cells stained positively with propidium iodine, suggesting again the dose of perforin used was most likely not sub lytic (37). Interestingly, when target cells were incubated with OT1 CTLs, on average, 10 pores ranged from 512 ± 49 nm to 370 ± 35 nm, which is consistent with the data obtained with purified perforin (37). Despite being very promising in terms of technical achievement, 4 hours of incubation with CTLs at an effector: target cell ratio of 20:1 alters the condition of the target cells and casts some reservations regarding the conclusions. It would have been very informative to perform AFM imaging after a shorter incubation time with the CTLs, which would coincide with the initiation of the lytic hit when perforin is delivering the granzymes. The wound healing model of perforin action supports that perforin makes small holes in the plasma membrane, resulting in a calcium influx, which initiates a wound healing response and rapid endocytosis of perforin and granzymes (38). Once inside the endosome, granzymes are released into the cytosol via a perforin-dependent mechanism (38, 39). The strength of this model relies on the fact that it is supported by evidence obtained both with purified native perforin as well as during effector cell conjugation, which is by far the most physiological approach. As stated earlier, it would be interesting to combine the live cell imaging used in this report with the AFM analysis to follow in parallel the occurrence of both the plasma membrane and endosomal pore formation. It is likely that they represent the extremes of the same process, depending on the level of perforin expression and discharge during cytotoxic lymphocyte attack. Nevertheless, regardless of the ongoing debate, collectively the different models all agree on the requirement of perforin for granzyme delivery, indicating that the induction of cell death is perforin dependent (40). Support for this notion is further strengthened by studies in perforin-deficient mice, which demonstrated the inability of cytotoxic lymphocytes to induce granule-mediated death or exhibit the correct release of granzymes from endosome vesicles in the absence of perforin (41–44). Interestingly, recent results indicate that cytotoxic lymphocyte kill trough the transfer supramolecular attack particles (SMAPs) composed of a cytotoxic core of perforin and GB wrapped in thrombospondin-1 shell of ~120 nm diameter in average that are originally stored in multicore granules (45). Interestingly, this SMAPs, are autonomously cytotoxic as they can kill target cells even after isolation from the cytotoxic lymphocyte (45). How these new findings fit the models of perforin mechanism is still not understood and will require more investigation. Perhaps taking advantage of the technology of alpha tagged perforin combined with highly specific anti-ALFA nanobodies could help answering to this question (46).

**Figure 2:**
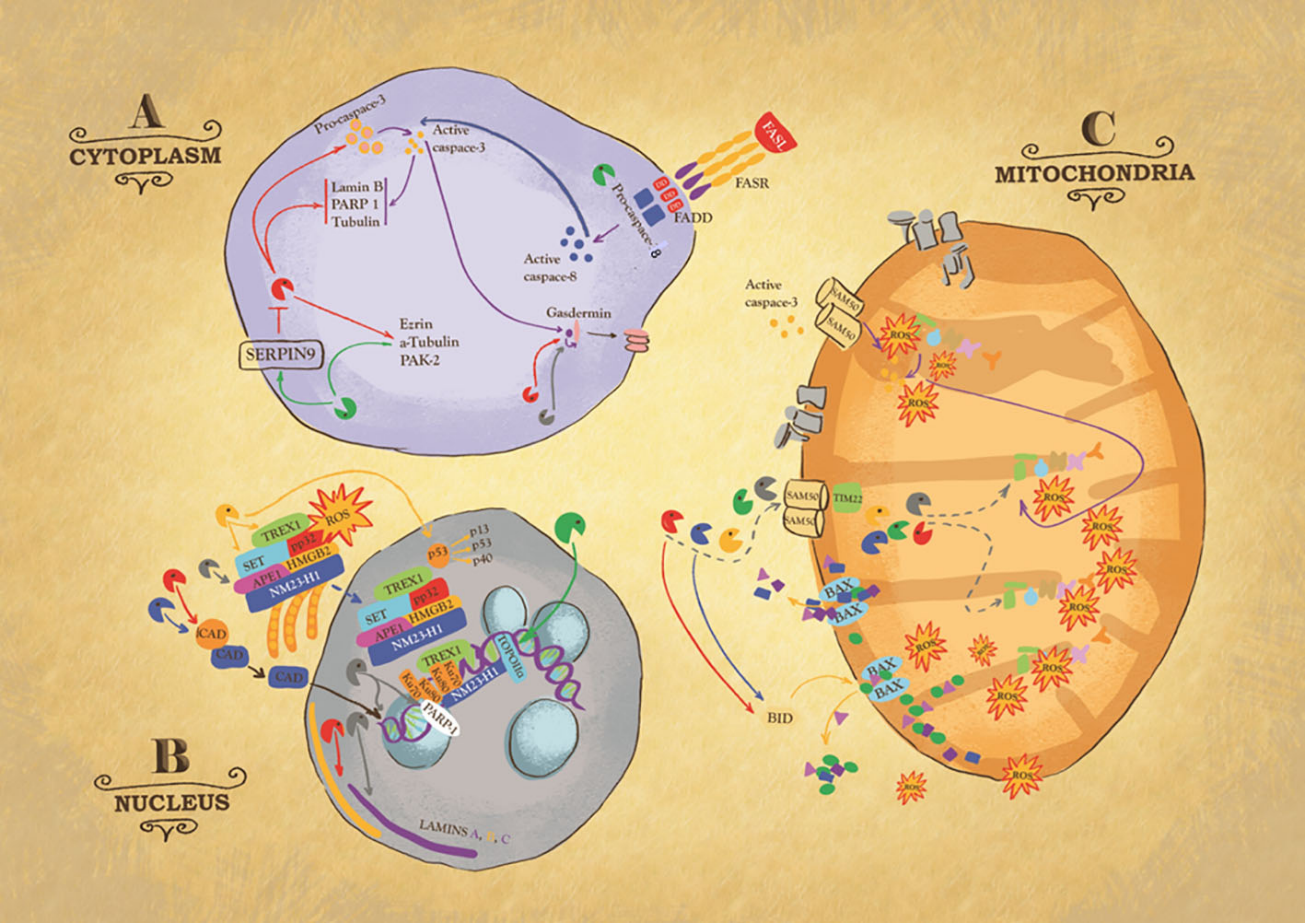
Intracellular action of granzymes. Action of granzymes in the cytoplasm **(A)**. Granzyme B (GB) can, directly or indirectly via the activation of procaspase 3, cleaves Laminin B, Nuclear Mitotic Apparatus protein (NuMa), Tubulin, Ezrin, p21 activated kinase 2 (PAK-2) and ICAD, inhibitor of the caspase-activated DNase (CAD), to trigger respectively caspase-independent or caspase-dependent cells death. Some of these substrates are also targets of Granzyme M (GM). GM also cleaves GB inhibitor SERPIN-9, suggesting its role in enhancing GB activity or targets FAS-associated death domain protein (FADD), causing the activation of caspase-8. GA, GB, and caspase 3 cleave member of gasdermin (GSDM) family, to trigger pyroptosis, a highly inflammatory type of cell death. Action of granzymes in the nucleus **(B)**. One critical target of GA is endoplasmic reticulum-associated (SET) complex. In presence of ROS, the SET complex is translocated from the endoplasmic reticulum to the nucleus, where its subunit SET, APE1 are cleaved by GA, leading to single stranded DNA nicking. Ku70 and PARP-1 proteins, which activate DNA repair machinery, are also targets of GA. GK also targets SET complex. GK cleaves p53, to produce cleavage fragments with a higher pro-apoptotic activity. GB and GH cleave ICAD, inhibitor of the caspase activated DNase (CAD), ensuring its translocation inside the nucleus where it breaks up DNA. GB and GA target laminin A, B and C disrupting the nuclear lamina. GM cleaves topoisomerase IIa. Action of granzymes in the mitochondria **(C)**. GB, GA, and GM enter mitochondria through the channels SAM50 in the outer membrane and TIM22 in the inner. It is likely that GH and GK use the same mitochondrial entry pathway. Once inside the organelle, GB and GA directly attack NDUFV1, NDUS1 and NDUFS2, subunits of mitochondria NADH: ubiquinone oxidoreductase complex I, causing the production of Reactive Oxygen Species (ROS). Importantly, GB causes mitochondrial outer membrane permeabilization (MOMP) by activating BH3-only protein (BID), which in turn triggers the oligomerization of Bax and/or Bak. Similarly, GH targets BID.

One characteristic feature of granzymes is their ability to induce target cell death through the activation of different, and sometimes redundant, pathways of cell death (47, 48) (**Figure 2**). By activating both apoptotic- and non-apoptotic death pathways, granzymes extensively contribute to the elimination of harmful damaged cells, reducing their dissemination, which highlights how the effectiveness of the immune system deeply relies on their regulated and effective release (16, 49). In the context of pathogen-infected cells, the ability of viruses, bacteria, and parasites to modify the cellular environment poses a serious limitation for the initiation of suicidal pathways and thus the activation of caspases (50–57). Therefore, the ability of granzymes to directly kill intracellular pathogens within infected cells in addition to killing the infected target cells is particularly important in the immune response to infection (58–60). It is then clear that the release of granzymes by cytotoxic lymphocytes is essential for eliminating infected cells that are unable to undergo apoptosis by themselves. Moreover, the combination of granzymes’ unique but complementary cleavage specificities further reduces the chances of immune evasion. To date, granzymes haven been shown to be involved in three distinct cell death pathways, spanning from caspase activation, mitochondrion alterations, and oxidative stress (8, 28, 61, 62). Among the five human granzymes, the pathways activated by GA and GB are the best characterized, although a growing body of evidence is finally revealing new roles for the so called “orphan granzymes” (61–66).

Like the caspases, GB cleaves after aspartic acid residues. Interestingly, the ability of GB to activate the caspases, concomitant with its capacity to also cleave specific apoptogenic molecules independently of the caspases, makes the GB-mediated death pathway both caspase dependent and independent (67, 68) (**Figure 2**). Among the first human and mouse GB substrates identified, pro-caspase 3 activation results in the cleavage of several cellular substrates involved in the apoptotic process. Although mouse GB is less efficient than human GB in activating BH3-only protein Bid, both mouse and human GB are capable of initiating Bid-dependent mitochondrial apoptosis in intact cells (13, 14). GB is responsible for the cleavage of Bid which disrupts mitochondrial membrane integrity by interacting with Bax and/or Bak, and for the cleavage of anti-apoptotic protein Mcl-1 (69–74). Studies *in vitro* have shown that both human and mouse GB is able to initiate the apoptotic cascade in the absence of caspase 3, although with less efficacy, by the direct cleavage of the inhibitor of the caspase-activated DNase (ICAD) (75, 76). Interestingly, several other intracellular substrates and cytoskeletal components were found to be targets of GB and GA cleavage, further potentiating the apoptotic response. At the nuclear level, lamin A, B and C, the main structural components of the nuclear lamina, are degraded by both human and mouse GB and GA before DNA fragmentation during apoptosis (77). Nuclear lamina is not the sole structural compartment targeted by granzymes. Indeed, human GB cleaves α-tubulin, while human and mouse GB cleaves ROCKII, thereby inducing cytoskeletal breakdown and plasma membrane blebbing (78, 79). GA cleaves after lysine and arginine and mediates cell death in a purely caspase-independent manner (**Figure 2**). However, the human and mouse GA cell death pathways still produce typical morphological features of apoptosis while avoiding the activation of executive caspases (65, 80). The endoplasmic reticulum-associated SET complex is critical to the human GA death program. Upon production of reactive oxygen species (ROS) triggered by human GA cleavage of NADH dehydrogenase iron-sulfur protein 3 (NDUFS3) from the mitochondrial complex I of the electron transport chain (ETC), the SET complex translocates into the nucleus, where it is cleaved by human GA, producing single-stranded DNA nicking (62, 81). Further cleavage of nuclear proteins Ku70 and PARP-1 inactivates the DNA repair machinery, while proteolysis of histone 1 linker and core histone tails makes the DNA even more accessible to nucleases (77, 82–84).

Along with the activation of caspases or the cleavage of specific factors, one important shared feature for both GA and GB is the production of ROS upon mitochondrial entry (61, 62, 66, 85–87) (**Figure 2**). At low intracellular doses, ROS are essential molecules involved in normal physiological cellular activities; however, when levels reach excess, they interfere with normal activities of the cell, causing damage to nucleic acids, proteins, and lipids. The strict relationship between ROS damage and granzyme activities is now well established. Human GA and GB can trigger mitocentric ROS-dependent apoptosis (61, 62, 66, 85–87). The increase in ROS levels contributes to apoptosis due to their role in the untethering of apoptogenic factors from mitochondria membranes, namely Endo G, smac and Cyt C, and in DNA fragmentation, as demonstrated by the delay in oligonucleosomal DNA fragmentation seen with ROS scavengers (66, 88, 89). The major source of ROS during the apoptotic process is the mitochondria; specifically, caspase- and granzyme-dependent cell death triggers disruption of ETC complex I (61, 66, 90). By directly attacking NDUFV1, NDUS1 and NDUFS2, all subunits of mitochondria complex I (NADH: ubiquinone oxidoreductase), GB triggers an increase in ROS, resulting in the disruption of the respiratory chain, loss of ETC complex activity and cristae junctions, finally compromising mitochondrial respiration (66). Similarly, GA also induces a rapid increase in ROS production and loss of transmembrane potential, causing nuclear translocation of the SET complex (61, 62, 66, 91). It is also worth noting that a study by Aguilo´et al found that mouse GB-triggered ROS production relies in part on a caspase-dependent-extramitochondrial ROS increase (92). In support of this conclusion, they showed that rho0 cells, which are depleted of mitochondrial DNA (mtDNA) and therefore no longer have a functional mitochondrial respiratory chain, still have normal ROS production following treatment with recombinant GB and sub lytic doses of streptolysin-O (SLO) used as a surrogate to perforin or used as target for GB-expressing mouse CTLs (92). They further showed that pretreatment with Z-VAD-fmk, the pan caspase inhibitor, or apocynin, a selective NADPH oxidase inhibitor, severely affects mouse GB-induced ROS production (92). This is in marked contrast with our results obtained with human GB. In the past, we often used Tiron, a super oxide scavenger. However, it was later found to have some direct effect on perforin function. Therefore, moving away from Tiron and using instead MnTBAP or MitoQ, two specific super oxide scavengers, the latter of which is mitochondrially targeted, we found that they both completely inhibit perforin and human GB treatment-induced ROS production and cell death (66). We also found that GB loading with perforin did not induce ROS production in K562 rho0 cells above the effect of perforin alone, and these cells were virtually completely resistant to GB-induced death. Importantly, scavenging the ROS or simultaneously overexpressing the non-GB-cleaved forms of NDUFV1, NDUFS1, and NDUFS2 reduced the ability of GB to trigger oligonucleosomal DNA fragmentation, apoptogenic factor release, and cell death (66). Importantly, GB could cleave these complex I subunits independently of mitochondrial outer membrane permeabilization (MOMP), as shown using Bax/Bak double knockout mouse embryonic fibroblasts (66). The fact that simultaneous overexpression of the GB-uncleavable forms of complex I subunits only partially protects from ROS and cell death could be explained by the fact that GB has many other cellular substrates, including other mitochondrial substrates, as suggested by GB disruption of the function of complex III and the mitochondrial cristae morphology (66). Indeed, it was also demonstrated that both killer cells and human GB induce a rapid stabilization and mitochondrial translocation of wild type p53 within target cells, which facilitate GB-induced MOMP (93). Moreover, pharmacological reactivation of nonfunctional mutant p53 improves GB and CTL and NK cell-dependent elimination of p53 mutated tumor cells (94). Our results agree with a previous report showing that caspase 3 also targets the mitochondrial complex I at the level of NDUFS1 to trigger ROS-dependent cell death (90). Since human GB can activate caspase 3, to some extent our results partly agree with Aguilo et al’s report on the contribution of caspase. However, our work demonstrates that GB can also act independently of the caspase, as it directly enters the mitochondria independently of MOMP, in a SAM50-dependent manner to directly cleave complex I subunits. Our data support a mitocentric ROS production, while those of Aguilo et al. favor an extramitochondrial NOX-dependent pathway. This discrepancy could be due in part to the difference between human and mouse GB. As stated above, granzymes from these two species do not completely functionally complement one another. It is also possible that the different models and approaches used, such as the type of rho0 cells, doses of inhibitors far above the IC_50_, and the genetic manipulation of GB mitochondrial substrates, could explain the different conclusions. Furthermore, human GB triggers MOMP by proteolytic cleavage and activation of the Bcl-2 family member Bid (13, 71, 95, 96). However, Bid-deficient mouse embryonic fibroblasts are still sensitive to GB-induced cell death, although they do not exhibit cytochrome c release from mitochondria (13). These results support the ability of GB to trigger ROS-dependent death either directly by entering the mitochondria or indirectly following caspase activation that cleaves many substrates, including NDUFS1, that are important for ROS-dependent death (85). The absence of a mitochondrial target sequence in both GA and GB has prompted us to investigate how these proteases enter the organelle. The canonical route for cytoplasmic-derive proteins into the mitochondria involves TOM40, the core channel of the translocase of the outer membrane and TIM23, the core channel of the inner mitochondrial membrane (97, 98). Still, we have demonstrated that both human GB and GA do not utilize this canonical entry route but require instead the SAM50 and TIM22 channels (85). These results suggest that cytotoxic molecules, like GA and GB but also caspases, that enter the mitochondria to cause damage might need to exploit alternative routes to overcome the mechanisms that maintain organelle integrity (85, 86). Interestingly, using NK cells deficient for GB it was shown that mouse GA triggers a caspase-, early mitochondrial alteration- and ROS-independent non-apoptotic cell death, albeit with slower kinetics than typical GB-dependent apoptosis (99). Cells undergoing this alternative cell death pathway termed athetosis have a writhing, ‘worm-like’ morphology, and mechanistically, this pathway relies on the actin cytoskeleton (99). These results agree well with an early report demonstrating the cytotoxicity of mouse GA and go further to bring interesting new mechanistic information regarding the strict requirement for a functional actine network (65, 100). However, this report did not reproduce the early mitochondrial alteration and ROS increase we observed in cells treated with sub lytic doses of perforin and recombinant human GA (61, 62). Actually, the cytotoxicity of human GA has been challenged by several reports, suggesting it has more of a pro-inflammatory function. However, although the role of granzymes (and not only GA) in the regulation of inflammation is well established, we still cannot deny the cytotoxicity of human GA. A fair review of our previous data would acknowledge that in addition to the questioned Tiron, we also used N-acetyl cysteine (NAC) and D1417 (3,5-dibromo-4-nitrosobenzenesulfonic acid) ROS scavenger, the latter of which is specific to superoxide, to show the ROS-dependence of the human GA pathway (62). Both NAC and D1417 protect target cells from GA- and perforin-induced ROS production and cell death, and also from GB-deficient as well as wild-type CTL-induced ROS production and cell death (62). Importantly, in a context where no recombinant or purified GA protease is present, we have shown that YT-Indy cells depleted of GB, GM, and GK with a combination of shRNAs targeting GB, GM, and GK, respectively, lose their cytotoxicity, which can then be rescued by re-expression of either wild-type human GB, GA, or GM (85). As stated earlier, we also showed that preventing GB, GA, or GM from entering the target cells’ mitochondria by silencing SAM50 protects from the killing mediated by YT-Indy expressing perforin with either GB, GA, or GM (85). Collectively, these results not only support that human GA is cytotoxic but also that mitochondrial alterations and mitochondrial ROS increases are essential in the GA pathway (85). One explanation for these results that conflict with other reports could be the quality of the different preparations of purified GA or the species of the purified enzyme. It is worth stressing that as stated earlier caspase 3 attacks the same respiratory chain complex I to cleave NDUFS1, while GA and GB cleave NDUFS1, NDUFS2, NDUFS3 and NDUFV1 of that same complex (61, 66, 90), and as we will develop later, granzymes tackle bacterial complex I and parasite homolog of complex I to trigger ROS-dependent microptosis (59, 60, 101). As Jacques Monod beautifully developed in his essay book, chance and necessity, this can’t be mere coincidence that three different cell death pathways target the same complex I of the respiratory chain across phylum. On the contrary these data rather highlight the importance of disrupting complex I for ROS production and cell death. It is also very likely that granzymes may have additional mitochondrial substrates to precipitated cell death, like for example Hax 1 (102). In agreement, with the importance of this pathway, we have further showed that preventing GA or GB and GM from entering the mitochondria protect them from cell death or significantly delay the cell death process by reducing apoptogenic factor release in the context of GB (85).

For a long time, the “orphan granzymes” were overshadowed by GA and GB, but now they have been shown to be not only important mediators of unique death pathways but also to act in synergy with the other granzymes to enhance their cytotoxicity (63) (**Figure 2**). Human GH cleaves after bulky, aromatic residues tyrosine and phenylalanine to trigger an alternative cell death pathway, sharing some similarity with GB, although the dependence on caspase activation, cytochrome c release, and the direct cleavage of ICAD and Bid remains unclear (23, 103, 104). Although the complete molecular signature is still debated, NK cells are prone to express GH, suggesting a putative role in infection clearance. Indeed, human GH cleaves 100K assembly protein and adenovirus DNA-binding protein (DBP), two non-structural proteins essential for the adenovirus life cycle and the latter also being a potent GB inhibitor (105–107). By cleaving 100K assembly protein, GH restores the cytotoxic activities of GB, which in turn activates apoptotic caspases that further cleave the same viral products, thus enhancing their antiviral effect (105–107). Human GK, like GA, has tryptase-like activity and substrates like those of GA, such as HMG2 and the SET complex components SET and ApeI, to trigger caspase-independent cell death and ROS production from mitochondria (108). Lymphocytes from GA-deficient mice express normal levels of GK and are still active against tumors, suggesting that GK can functionally compensate for the absence of GA and act in synergy with GB (100, 109). However, human GK differs from human GA in its ability to cleave Bid to induce cytochrome c and endonuclease G release from the mitochondria (110). Human GM, which cleaves after methionine or leucine, is characterized by a more unique type of death pathway that phenotypically resembles that of necrosis and autophagy (111). Human GM directly targets α-tubulin, ezrin, apoptosis-related p21-activated kinase 2 (PAK-2) and CAD, some of which are also targets of GB, although cleavage by these two proteases occurs at different sites and with different kinetics (112, 113). Furthermore, the ability of GM to cleave and inactivate GB SERPIN-9 inhibitor indicates the possible involvement of GM in enhancing GB activity (114).

### 2.2 Cytotoxic and non-cytotoxic functions in tumor eradication

Pre-malignant and transformed cells represent a serious threat to the organism. During immune surveillance, the immune system scrutinizes the entire organism to eliminate such threats as they emerge (115–117). Again, granzyme-mediated cell death induced by CTLs and NK cells represents a predominant and powerful strategy for effectively eliminating these dangers (115). This is further supported by the fact that perforin-deficient mice are more prone to develop spontaneous and aggressive disseminated B-cell lymphoma and fail to efficiently reject many transplanted tumors (42, 118, 119). Hypomorphic *perforin* mutations, which do not completely alter perforin function, are associated with late-onset of FHL, lymphoma, and other cancers (120). However, it is noteworthy that in addition to intracellular substrates directly involved in cell death induction, as discussed earlier, other granzyme substrates are involved in multiple steps of tumor initiation, progression, and dissemination (**Figure 2**). For example, all human granzymes cleave heterogeneous nuclear ribonucleoprotein K (hnRNPK), reducing tumor cell viability (121). Similarly, GM also targets topoisomerase IIα and FAS-associated death domain protein (FADD), resulting in its dimerization and activation of pro-caspase 8 (121). Cleavage of p53 by human GK, albeit apparently a pro-tumoral process, was found to be particularly effective against tumor cells due to the strong pro-apoptotic activities of the three p53 cleavage products, which therefore enhance GK-mediated tumor killing (122). Similarly, GH efficiently kills tumor cells after perforin- or streptolysin 0-mediated delivery by achieving typical hallmarks of programmed cell death (103).

Recently, the multiple death pathways triggered by granzymes were extended to pyroptosis. This type of cell death, mediated by caspase 1, is linked to the disruption of extracellular or intracellular homeostasis associated with innate immunity (123). It took several years to finally identify gasdermin D (GSDMD) as the effector molecule involved in this type of cell death (124–126). The gasdermin family of proteins possess inherent necrotic activity in their N-terminal domain, which is kept inactive by the inhibitory gasdermin C-terminal domain (126, 127). Upon proteolytic cleavage, the gasdermin N-terminal domain is translocated into the plasma membrane where it oligomerizes to form large 10–15-nm diameter pores, allowing for the secretion of inflammatory molecules and leading to cell swelling (126, 128, 129). This type of non-apoptotic cell death pathway is gaining increasing interest in the context of cancer treatment, prompting the quest for drugs, reagents and natural products that can trigger pyroptosis (130, 131). Recently, human GA and GB were shown to trigger pyroptosis in gasdermin B- and E-expressing cells, respectively (132–134) (**Figure 2**). Caspase 3 also triggers activation of gasdermin E in the elimination of chemotherapy-treated cells or vesicular stomatitis virus-infected cells (135, 136). Interestingly, cancer cells were shown to accumulate loss-of-function mutations in the gasdermin E gene or to epigenetically silence receptor-interacting protein kinase-3 (RIPK3) gene expression, the latter being an essential mediator of necroptosis (132, 137). These results suggest that during tumor development and progression, there is a selection for the loss of immunogenic cell death mediators such as GSDM or RIPK3 (132, 137). Collectively, these results further underline the center role of cytotoxic lymphocytes in the eradication of cancer. It is therefore not surprising that novel immunotherapy strategies, such as chimeric antigen receptor (CAR) T-cell or CAR-NK cell adoptive transfer immunotherapy, focus on redirecting the killing power of these lymphocytes onto the target cancer cells (138). However, excessive pyroptosis could result in the detrimental cytokine-release syndrome (CRS), a serious side effect of CAR T-cell adoptive transfer immunotherapy in the context of hematologic malignancies that co-express gasdermin (134).

### 2.3 Non-cytotoxic anti-microbial functions

The direct antimicrobial activities of granzymes have opened the door to gain new insights in their intracellular roles, primarily their ability to target pathogenic entities rather than the infected cell (139, 140). An interesting example is human adenovirus type 5, which is known particularly for immunomodulatory infection. The E1, E3 and L4 adeno-transcription units encode for proteins able to interfere with both the canonical recognition of infected cells and their elimination. As previously stated, human GH cleaves 100K assembly protein, a direct inhibitor of GB, to restore the cell death induced by cytotoxic lymphocytes (107). Mouse GB also was shown to directly cleave the immediate early protein ICP4 to control herpes simplex virus-1 latency (141). Similarly, human GM cleavage of human cytomegalovirus phosphoprotein 71 interferes with viral replication by limiting the expression of immediate early viral genes (142). However, because of the redundancy of their pathways and their ability to cleave specific viral proteins, the granzyme family still represents a potent response of the body against infection. In addition to viral components, many host proteins with no direct apoptogenic roles but that contribute to the viral life cycle were found to be targets of granzyme cleavage. These factors actively participate in multiple steps of virus life cycle such as viral DNA replication, transcription, translation, viral assembly, and retroviral DNA integration (143–145).

Importantly, the active roles of granzymes are not restricted to virus-infected cells. A growing body of evidence suggests that granzymes may play an important role in inducing bacterial or parasitic cell death in a granulysin- and perforin-dependent manner *in vitro* (60, 143–146). These reports highlight the essential contribution of ROS production in the elimination of such entities. Human granzymes not only trigger ROS release through their action on the mitochondrial ETC, as previously described in this review, but by directly targeting the pathogens ETC complex I and antioxidant defense mechanism, mostly in relation to superoxide dismutase and catalase, they directly affect the viability of these pathogens (60, 101). The production of high amounts of ROS during granzyme-mediated cell death was demonstrated to be toxic by itself, causing direct damage to the pathogen, and also to enhance apoptotic processes, i.e., DNA damage, membrane lipid and protein oxidation (60, 101, 146). *Trypanosoma cruzi, Toxoplasma gondii* and *Plasmodium falciparum* were found particularly susceptible to this type of oxidative stress. This results in morphological alterations and decrease in MMP, DNA oxidation, chromatin condensation, mitochondrial swelling, plasma membrane blebbing and phosphatidylserine exposure (58, 59). *In vitro*, granzymes actively take part in the induction of bacterial or parasitic cell death in a granulysin- and perforin-dependent manner (60). *In vivo*, while the deletion of GB did not affect the outcome of the bacterial killing in mice, this could be explained by the potential of other intact granzymes, such as GA and GM, to cleave bacterial substrates and thus control the infection (60). Moreover, in another *in vitro* study, GA was shown to opsonize Mycobacterium tuberculosis to limit monocyte infection (147). All in all, granzymes are very likely to play an important role in the immune system’s ability to fight infections, and future studies should focus on establishing this defense mechanism *in vivo*.

## 3 Pathogenic roles of granzymes: the bad and the ugly

### 3.1 Granzymes in immune regulation and immunosuppression

The ability of malignant cells to avoid immune recognition and/or elimination as a result of the activation of immune-escape strategies still represents a serious obstacle in cancer treatment (148). Unfortunately, some of the tactics the immune system uses to eradicate cancer cells are used by cancerous cells to facilitate their growth and dissemination (149). Accumulated evidence suggests that granzymes also participate in tumor escape by contributing to the formation of an immunosuppressive environment as well as by eliminating effector cells (150). This process that is particularly relevant in the tumor microenvironment (TME) where TIM-3^+^ T regulatory cells (Tregs) demonstrate higher cytolytic activity toward autologous T conventional cells compared to TIM-3^-^ Tregs (29, 150, 151). Importantly, under proper stimulation or even constitutively, granzymes can be expressed by bystander cells present in the TME (29). Tregs and myeloid-derived suppressor cells (MDSCs) mediate immunosuppression of the anticancer response by eliminating NK cells and CD8^+^ T cells in a GB- and perforin-dependent manner (152, 153). Tregs represent a subpopulation of T cells that physiologically maintains tolerance in the periphery. Unfortunately, these cells are also associated with tumor evasion and metastasis via their role in suppressing anti-tumor immunity (152, 154). Relevant Treg activities span from inhibiting cell functions to inducing apoptosis upon the release of GB in antigen-presenting cells, B cells and T effector cells (151, 155). MDSCs are another interesting example, as they can negatively affect dendritic cells, macrophages, T cells and NK cells, by producing excessive levels of ROS and arginase I (156–158). However, a contact-dependent inhibition of T lymphocytes by MDSCs was noticed in recent research, demonstrating that perforin and GB found in these cells act as effector molecules to promote tumor progression, mainly by exploiting their cytotoxic roles towards effector T cells (153). Moreover, granzyme expression is observed in many cell types other than classical cytotoxic CD8^+^ T cells and NK cells. For instance, granzyme but not perforin expression has been found in B cells, macrophages, mast cells, basophils, and plasmacytoid dendritic cells (pDCs) (150, 159–165). Note, Kupffer cells, the dominant liver resident macrophages express granzyme and perforin, suggesting that the expression of these cytotoxic proteins may vary within the same cell type dependent on the tissue of residence (166). After its secretion, GB can indiscriminately kill cells in its immediate environment, but the underlying mechanism is not fully understood. It was also proposed that extracellular GB can adsorb to bystander cells prior to being endocytosed to trigger cell death via uncharacterized mechanisms (150). Another possibility is the triggering of anoikis as demonstrated in the case of GB secreted by mast cells (162). Regulatory B cells (Bregs) are a subpopulation of B cells with a regulatory phenotype and inhibitory activity towards other anti-tumor cells (167). These cells express inhibitory ligands, including programmed death-ligand 1 (PD-L1), and secrete the anti-inflammatory cytokines interleukin-10 (IL-10) and transforming growth factor beta (TGF-β), which further enhance the differentiation of Tregs (150). By exploiting the pro-apoptotic features of GB, Bregs, similarly to Tregs and pDCs, inhibit T-cell responses and trigger non responsiveness of the immune cells, suppressing the proliferation of CD4+ T cells, NK cells, B cells and other bystander cells (159). Unlike Tregs and Bregs, pDCs are not always involved in immunosuppression. During innate immunity, they play an important role by secreting type I interferons (IFNs) in response to pathogen recognition by Toll-like receptor, and they can participate to anti-tumor immunity through the activation of antigen-specific T cells (168). However, in a context-specific manner, pDCs’ interaction with tumor cells may also lead to immunological tolerance (169, 170). In addition, pDCs’ paracrine release of GB into the extracellular milieu may facilitate tumor invasion, inhibiting T cell function by cleaving the CD3ƺ chain (171).

Interestingly, granzyme but not perforin expression has also been observed in non-immune cells, such platelets, human articular chondrocytes and even cancer cells, while in the case of keratinocytes, both granzyme and perforin expression was recorded (172). Many tumor types, both solid and liquid, show expression of several types of granzymes, suggesting possible roles for granzymes in tumor initiation and development. For example, GB was detected in human primary breast carcinomas, primary bladder tumors and pancreatic carcinoma cells, whereas GM expression was found in T cell granular lymphocytic leukemia cells and in the HeLa cell line (173–175). Collectively, these results indicate that granzymes may confer these tumor cells with some growth advantage through mechanisms that remain to be characterized.

### 3.2 Granzymes in inflammatory and chronic diseases

#### 3.2.1 Extracellular activities

**Figure 3:**
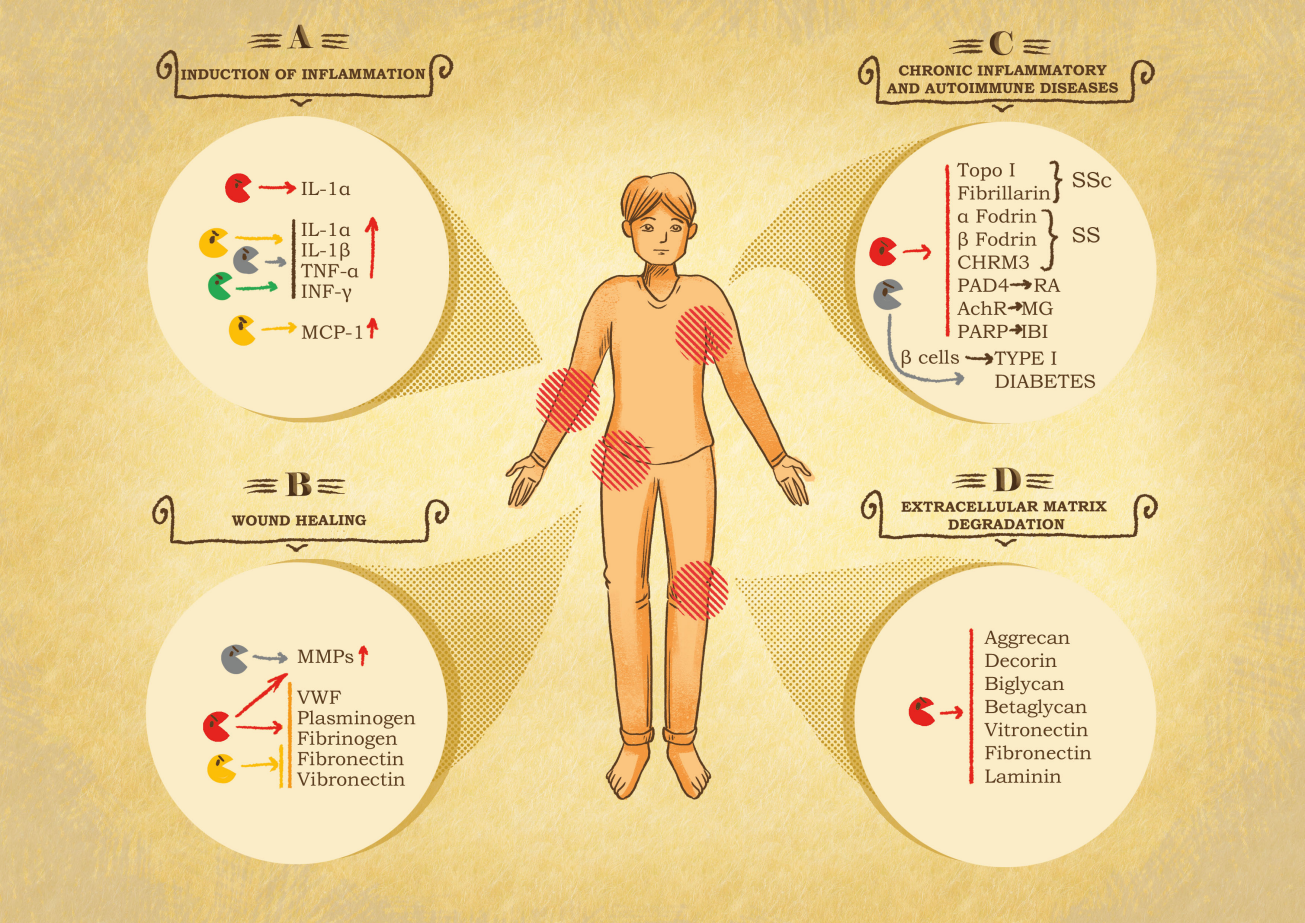
Role of granzymes in pathological settings. **(A)** Inflammation induction. After leakage from the immune synapse, granzymes can act on the immediate surrounding to potentiate the immune response at the cost of exacerbated inflammation and therefore inflammatory diseases. GB cleaves IL-1α, producing a more active fragment. GA, GK and GM internalization and catalytic activity trigger the secretion of pro-inflammatory cytokines, e.g., IL-1α, IL-1β, TNF-α and INF-γ by immune system cells. GK triggers the production of monocyte chemoattractant protein 1 (MCP-1) by pulmonary fibroblast. Chronic inflammation linked to GB, GA and GK activity has been shown in the onset of rheumatoid arthritis (RA), spinal cord injuries and in chronic obstructive pulmonary disease (COPD). **(B)** Chronic wound healing. Granzymes can impair or delay provisional matrix formation, angiogenesis and re-epithelization following the cleavage of von Willebrand factor (VWF), plasminogen, fibrinogen, fibronectin and vitronectin and stimulates metalloproteases (MMPs) in fibroblasts, that further exacerbate tissue damage. **(C)** Systemic autoimmune diseases. Granzymes have been associated with autoimmune disease and cardiovascular, pulmonary, and neurological disorders due to their catalytic activity, resulting in the production or modification of autoantigens. In this context GB is the main actor, cleaving autoantigens involved in scleroderma (SSc) Sjögren syndrome (SS), RA, myasthenia gravis (MG), and ischemic brain injuries (IBI). GA contributes to diabetes type I, due to its participation in pancreatic β-cell degradation. **(D)** Extracellular matrix degradation. Protein components of the extracellular matrix are target of granzymes, resulting in the restriction of tumor invasion, tissue destruction, neurotoxicity, and autoimmune or inflammatory diseases. GB cleaves proteoglycans such as aggrecan, decorin, biglycan and betaglycan, which compromises the integrity and organization of the matrix. Similarly, GB also digests adhesive proteins such as laminin, fibronectin and vitronectin, essential components in the skin tissue, therefore contributing to skin damage and pathological conditions.

As emphasized earlier, granzymes also have extracellular functions. By shifting the research focus from their pro-apoptotic to pathological roles, it was possible to link the elevated levels of granzymes in bodily fluids to their participation in disease settings (**Figure 3**). In healthy individuals, serum levels of granzymes are typically low (i.e., median healthy GB levels range from 20–40 pg/mL) (176, 177). Elevated levels of circulating granzymes are found in several disease conditions, most commonly during infections, acute inflammation, and autoimmune diseases. GB is present in synovial and cerebrospinal fluids of respectively rheumatoid arthritis and multiple sclerosis (MS) patients while it is also present in the bronchoalveolar lavage from chronic obstructive pulmonary disease (COPD) patients, with GB expressing CD8^+^ T cells directly correlated with progression and disability in MS (28, 159, 178–182). Although the molecular mechanism of granzyme discharge in the extracellular space is still obscure, it is estimated that about one-third escapes the synapse. Possible reasons for this secretion are T-cell receptor stimulation or prolonged exposure to IL-2, degranulation after engagement with extracellular matrix (ECM) proteins or inflammatory cytokine production upon lipopolysaccharide (LPS) or bacteria recognition (176, 183). Another important mechanism involves cells such as mast cells, which upon stimulation directly degranulate their granule content and secret granzyme (162). Like their intracellular functions, the extracellular roles and substrates are better characterized for GB and GA than for the orphan granzymes.

#### 3.2.2 Induction of inflammation

Both human and mouse GB cleavage of IL-1α was observed during NK cell-mediated killing, resulting in IL-1α fragments with more potent activity than the full-length cytokine (184) (**Figure 3**). Similarly, regulation of cytokine expression is also observed for human and mouse GA (180). Physiologically, during infection, a modest leak of GA from the immune synapse can activate local monocytes or recruit them to the site, which serves to maintain an effective level of local inflammation where it is needed. Both GA internalization and catalytic activity are required for increased cytokine production and release from monocytes (180, 185, 186). Interestingly, human GA-dependent cytokine cleavage provides an inflammasome-independent alternative pathway for pro-inflammatory cytokine activation (187). In agreement with granzymes’ contribution to inflammatory cytokine production, it was further shown that like GA, mouse GM is involved in the production of IL-1α, IL-1β, tumor necrosis factor alpha (TNF-α) and INF-γ following LPS-Toll-like receptor 4 (TLR4) signaling (188). Likewise, GK triggers pro-inflammatory cytokine release from monocytes, human lung and skin fibroblasts and keratinocytes in a protease-activated receptor 1 (PAR-1)– and ERK1/2-dependent manner (161, 189, 190). Furthermore, monocyte chemoattractant protein 1 (MCP-1) release is increased upon human GK treatment of pulmonary fibroblasts, highlighting the role of GK in stimulating inflammation through the recruitment and activation of immune cells (191). Whether granzyme-mediated cytokine release is exclusively linked to the novel role of granzymes in the activation of the gasdermins is an open question. Tight regulation of extracellular granzyme activity is essential for precise control of pro-inflammatory cytokine responses, as its dysregulation leads to inflammatory diseases. This is illustrated by the involvement of GA-induced cytokines in gut inflammation and colorectal cancer development (192).

#### 3.2.3 Cleavage of extracellular receptors and ECM substrates

Like caspases and metalloproteases, granzymes also cleave intracellular and extracellular receptors such as the thrombin receptor (193, 194). Cleavage of these substrates, due to their relevance in cell proliferation, cell differentiation, ECM remodeling, vascularization, and cell migration, could have important implications in disease settings (193, 194) (**Figure 3**). The functional consequences and relevance of these cleavage events are still not clearly understood, although research suggests they may induce receptor activation, signaling, apoptosis or other cellular processes (31). For example, cleavage of PAR-2 by trypsin and GA is thought to induce pro-inflammatory effects, as its activation induces IL-8 and granulocyte-macrophage colony-stimulating factor (GM-CSF) expression in keratinocytes (195, 196). GA cleavage of thrombin-like receptor on neurites induces neurite retraction and reverses stellation of astrocytes, whereas PAR-1 activation by GB is neurotoxic and contributes to brain atrophy (197). GK is better able to cleave PAR-1 to trigger the proliferation of fibroblasts and their cytokine production (189, 198). Human GB cleavage of the glutamate receptor (GluR3) from the surface of CTLs results in reduced T-cell adhesion to laminin, thus leading to altered cross talk between T cells and nerves in neurological and autoimmunity diseases (199). Interestingly, GB only cleaves the non-glycosylated form of GluR3, indicating that alterations in post-translational modifications can induce uncommon cleavage products (29).

In addition to cellular receptors, protein components of the extracellular environment also have been found to be targets of granzymes (**Figure 3**). Proteolysis of ECM results in restriction of tumor invasion, tissue destruction, exacerbated autoimmune or inflammatory arthritis as well as neurotoxicity (176, 200). Only recently was the significance of ECM targeting by human granzymes recognized and the direct link to certain pathological conditions discovered (161, 172). Abundant targets have been identified in the skin tissue, and so far, these targets are mostly associated with skin conditions, such as aging, scarring, wound repair and fibrosis (30). Indeed, laminin, fibronectin and vitronectin are all substrates for human GB-mediated cleavage (172). These adhesive proteins are abundant in the junction between derm and epidermal where they are essential for attachment and crosstalk between these two layers. Consequently, their cleavage induces skin tearing, reduced nutrient exchange and diminished integrity in aged skin (29). Human and mouse GB also contributes to skin aging and diseases by degrading aggrecan, decorin, biglycan and betaglycan proteoglycans, which determine the correct organization of the matrix for the proper maintenance of tissue structure, porosity, and integrity (30, 172). In healthy skin, decorin inhibits excessive collagen deposition to limit fibrosis (201, 202). Consequently, the degradation of decorin by GB disorganizes collagen, reduces tensile strength and decreased growth factor and cytokine deposition in the altered ECM (29, 202). Not surprisingly, GB is now used as a therapeutic target to treat inflammatory skin condition such as atopic dermatitis (AD) (203). The contribution of GB in autoimmune skin conditions has recently been reviewed by Gleave, A. et al. (204). Similarly, GK by cleaving the endothelial cells glycocalyx component syndecan-1 triggers microvasculature damage and exacerbate AD (205). The role of GK expressing CTL in autoimmunity has been reviewed by Jonsson A. H. Recently, transcriptomic analysis has identified GK expressing CTL as novel biomarker of Alzheimer’s Disease suggesting an involvement in the etiology or the progression of the disease.

#### 3.2.4 Granzymes in chronic wound pathogenesis

Wound healing is a critical process for restoring proper skin and tissue homeostasis, and it occurs via four phases: hemostasis, inflammation, tissue regeneration and tissue remodeling (206). Considering that all these phases involve ECM proteins, which are also direct GB substrates, excessive degradation in these components readily affects the entire healing process, resulting in a chronic wound pathogenesis (30) (**Figure 3**). The hemostasis process is negatively affected by granzyme-mediated cleavage and inactivation of von Willebrand factor (VWF), plasminogen and fibrinogen, which are all crucial to the clotting cascade (200, 207). GB cleaves an essential domain in VWF, preventing platelet aggregation and producing antiangiogenic fragments by cleaving plasminogen at alternative sites (200). Consequently, GB alters the angiogenesis process and delays wound healing, as seen in patients with scleroderma (207). The re-epithelialization process can be further delayed by GB-mediated cleavage of fibronectin and vitronectin, which results in the destruction of a provisional matrix necessary for the migration and proliferation of keratinocytes across the wound bed (208). Impaired re-epithelization was also observed in the presence of GK during thermal injury-related wound healing (161). Keratinocyte exposure to granzymes results in decreased cell migration, possibly through the induction of the expression of pro-inflammatory cytokine and ECM degradation (209). Finally, GB- and GA-mediated activation of improper inflammatory cytokine release induces a pro-inflammatory environment that stimulates metalloprotease production by fibroblasts, which in turn exacerbates tissue damage and thereby further delays the wound healing process (30).

#### 3.2.5 Granzymes in chronic inflammatory and autoimmune diseases

Increasing evidence supports roles for granzymes in autoimmune diseases, cardiovascular and pulmonary conditions and neurological disorders (30). Systemic autoimmune diseases represent a group of complex disorders that lead to different clinical outcomes arising from the immune reaction to self-antigens (30, 210). Traditionally, apoptosis has been recognized as the crucial initiator of this condition, due to the accumulation of cell autoantigens in apoptotic bodies, which are then captured and presented to the immune system (211). Based on the active participation of granzymes in the apoptotic cascade, it was hypothesized that granzyme activities could be involved in autoantigen production or modification (210) (**Figure 3**). Indeed, alterations of protein conformation following post-translational modifications, change in pH or physical force are likely to unmask hidden cleavage sites, consequently revealing cryptic epitopes or dominant epitopes, which in both cases causes the immune system to recognize the self-antigen (29, 210). In support of this hypothesis, 21 of 29 well-described autoantigens for systemic lupus erythematosus, scleroderma and Sjögren syndrome were found to be targets of GB cleavage (29, 212). Importantly, these results were further illustrated *in vivo* by the particularly high frequency of finding GB-generated autoantigen in the sera of patients affected with ischemic digital loss in systemic sclerosis (212, 213). In certain patients, GB cleaves several systemic sclerosis autoantigens, such as topoisomerase I and fibrillarin, which induces ischemic digital loss, a disease complication (212, 213). In Sjögren syndrome, CD8^+^ and CD4^+^ cells induce apoptosis in epithelial cells through a granzyme/perforin pathway in which GB cleaves α-fodrin, β-fodrin, type 3 muscarinic acetylcholine receptor and La autoantigen (214–216). GB has also been associated with rheumatoid arthritis, an organ-specific autoimmune inflammatory disease that results in joint and tendon tissue destruction (29). Extracellular GB levels are extremely high in the synovial fluid and plasma of rheumatoid arthritis patients, suggesting that infiltrating CTLs secrete extracellular GB, thus promoting ECM degradation and remodeling (177, 217). These GB and GA substrates induce neutrophil and monocyte infiltration, activation of metalloproteases and disruption of chondrocyte cell adhesion (176). The GB-mediated ECM degradation further facilitates the migration of cytotoxic T cells and mononuclear cells through the endothelial basement membrane contributing to hyperplasia and destruction of the joint (218). Some evidence indicates the presence of GB-derived rheumatoid arthritis autoantigens; specifically, peptidyl arginine deiminase 4 (PAD4) was able to promote enhanced stimulation of autoreactive T cells, thus contributing to rheumatoid arthritis pathogenesis (219). An increased level of GA has also been found in the plasma, the synovial fluid, and tissue biopsies of rheumatoid arthritis patients (177, 220). Importantly, GA might be implicated in the augmentation of local inflammation via stimulation of pro-inflammatory cytokine release (9, 217). Furthermore, GA contributes to the differentiation of mouse osteoclast, cells that contribute to joint destruction in rheumatoid arthritis (221). The role of granzyme in rheumatoid arthritis has recently been reviewed by Zheng, Y. et al. (222). Autoimmune type I diabetes is another condition in which granzyme-mediated apoptosis plays a critical role. Indeed, pancreatic β-cell loss and the resulting insulin insufficiency are direct consequences of the reactivity of T cells to one or more β-cell autoantigens (223–225). Interesting, a contribution of GA to β-cell loss has been demonstrated (226). Finally, GB has also been implicated in the pathogenesis of neurological disorders (29). In spinal cord injuries, elevated counts of CTLs and levels of GB and pro-inflammatory cytokines were found in proximity to damaged neurons (29). In ischemic brain injury, the increased production of ADP ribose polymerase fragments, known products of both GA and GB cleavage, suggests a crucial role for such cleavages in inducing neuronal death, which then contributes to the pathophysiology of this condition (83, 227, 228). Similarly, GB cleaves the acetylcholine receptor ϵ (AChR-ϵ) subunit, suggesting it may contribute to myasthenia gravis (MG) (229). The implication of GB in MG is further supported by the finding of GB in the thymus of MG patients but not in that of controls (229). By cleaving the AChR-ϵ subunit, GB would expose cryptic antigens, which in turn induces the autoimmune response and reduction in AChR functionality (229). Of course, additional experiments are needed to clearly demonstrate the direct cause and effect of GB action in MG pathogenesis.

The ability of granzymes to induce cell death other than apoptosis, such as anoikis, also contributes to the development or progression of certain pathological conditions. This specific type of cell death is caused by a loss of proper cell–matrix interactions, and physiologically, it is meant to avoid abnormal growth and adhesion on improper ECM (230). In both physiological or pathological contexts, GB induces cell detachment by cleaving essential matrix components (231). While this might represent an important process for appropriate tissue renewal, in the context of diseases, excessive cell death and tissue injury lead to severe complications (231). In several acute pulmonary pathologies, including COPD, the density of infiltrating CD4^+^, CD8^+^ and NK cells directly correlates with amount of bronchial epithelial cell apoptosis and/or anoikis, in agreement with alveolar ECM collagen IV, fibronectin and laminin being direct GB substrates (232, 233). Collectively, with the elevated levels of GB in bronchoalveolar lavage fluid, these results suggest that granzymes may, intracellularly, induce cell death, and extracellularly, induce alveolar wall damage and anoikis (234). GA levels are also increased in lung tissue of COPD patients, where like GB, GA causes extensive ECM degradation as well as epithelial cell rounding and detachment (235). The pro-inflammatory roles of GA are correlated with increases in IL-6 and IL-8 expression in COPD patients, probably though the disruption of microtubule structures (236). Overall, this evidence supports a role for GA in the pathology of lung disease, due to the induction of inflammation, loss of alveolar wall structure and neutrophil infiltration (237). Notably, the GK level also was found to be increased in COPD (178). In this setting, GK-induced enhanced inflammation, along with increased IL-6, IL-8, and MCP-1 expression, contributes to airway remodeling (189).

New putative roles for GB have emerged in atherosclerosis, a lipid-dependent inflammatory condition at the etiology of most heart attacks and strokes (238, 239). Here again, T lymphocytes and macrophages participate in atherosclerosis, and an elevated GB level is found in the plasma of atherosclerosis patients (240). This evidence supports the hypothesis that GB can influence plaque stability, probably through ECM destruction, release of the trapped cytokines and induction of anoikis of smooth muscle and endothelial cells in human coronary arteries (241, 242).

Lastly, because GA, GB, GK, and GM have proinflammatory functions, it was hypothesized that they are likely to contribute to the pathophysiology of sepsis (180, 243–245). In agreement with this possibility, recent studies have observed that circulating levels of GA, GB, GK, and GM are significantly increased in the conditions of endotoxemia and sepsis, although this was not true for GA in the condition of sepsis in a burn patient (246–251). These initial observations were further supported by the fact that mice deficient in GM and GA not only survived better after experimental endotoxemia, but they expressed lower levels of proinflammatory cytokines (180, 188). Interestingly, in experimental human endotoxemia, injection of LPS in healthy volunteers triggered a transient increase in circulating GM, while the effect on circulating GK was milder (247). Noteworthy, only *Pseudomonas aeruginosa* stimulation of whole blood could trigger the release of GK, suggesting that not all gram-negative bacteria are equal inducers of GK secretion (247). Interestingly there is a direct link between inflammation and coagulation. Indeed, inflammatory cytokines can activate clotting while reducing the anticoagulant system and dysregulating the fibrinolytic system, which are dysregulated in sepsis (248, 252). Note that GB and GM, independently of their ability to affect cytokine levels, can directly cleave Von Willebrand factor to limit its binding to factor VIII and modulate the coagulation process (253). This novel function of granzymes in endotoxemia and sepsis has been recently reviewed by Garzón-Tituaña et al. (248). Collectively, these results suggest a complex role of soluble granzymes in the pathophysiology of sepsis, although the molecular mechanism at play requires further characterization. Moreover, in line with the increasingly important role of granzyme in sepsis, it is also important to identify the origin of the secreted granzymes. Are they produced by a wide array of cell types or is a certain lineage largely responsible for the secretion of granzymes in the context of a specific strain of gram-negative bacteria, as shown for GM and GK (247)?

## 4 Conclusions

Granzymes, a family of serine proteases, are powerful pleiotropic enzymes involved in various physiological as well as pathophysiological processes. Their essential roles in the elimination of malignant or pathogen-infected cells underline their prominent place in the proper functioning of the immune system. While the induction of apoptosis was believed to be their only activity, and GA and GB were considered the main mediators of this function, additional evidence has shown that granzymes can directly interfere with viral replication, bacterial growth, and tumor growth independently of cell death. Research has also shown that granzymes accentuate local inflammation and are active outside the cell. Orphan granzymes have been recognized as essential mediators of these processes along with GA and GB. Furthermore, the observations that granzyme concentrations are increased in certain pathological conditions and that they are also expressed in non-immune cells, has laid a foundation to re-evaluate the initial view of these enzymes as mere apoptosis inducers. Their unregulated release, expression in immunosuppressive cells, cleavage of disease-related substrates, and finally, exacerbation of the inflammatory process are just some of the mechanisms identified to date. Moreover, recent exciting results implicated granzymes in pyroptotic cell death. Collectively, these recent developments have identified granzymes and their inhibitors as promising potential therapeutic targets.

## Author contributions

DM: Conceptualization, Project administration, Supervision, Validation, Writing – original draft, Writing – review & editing. LC: Conceptualization, Data curation, Investigation, Validation, Writing – original draft, Writing – review & editing.
